# Successful surgical approach for a patient with encapsulating peritoneal sclerosis after hyperthermic intraperitoneal chemotherapy: a case report and literature review

**DOI:** 10.1186/1471-2482-14-57

**Published:** 2014-08-27

**Authors:** Katsushi Takebayashi, Hiromichi Sonoda, Tomoharu Shimizu, Hiroyuki Ohta, Mitsuaki Ishida, Eiji Mekata, Yoshihiro Endo, Tohru Tani, Masaji Tani

**Affiliations:** 1 Department of Surgery, Shiga University of Medical Science, Seta, Tsukinowa-cho, Otsu, Shiga 520-2192, Japan; 2 Department of Clinical Laboratory Medicine, Shiga University of Medical Science, Seta, Tsukinowa-cho, Otsu, Shiga 520-2192, Japan

**Keywords:** Hyperthermic intraperitoneal chemotherapy, Encapsulating peritoneal sclerosis, Sclerosing encapsulating peritonitis

## Abstract

**Background:**

Encapsulating peritoneal sclerosis (EPS) is a rare surgical complication that can occur after intraperitoneal treatment. It is also a serious and potentially fatal complication of continuous ambulatory peritoneal dialysis. The present report describes a case of surgically treated EPS that probably occurred as a complication of hyperthermic intraperitonal chemotherapy (HIPEC).

**Case presentation:**

A 39-year-old man required sigmoidectomy for serosal invasive advanced sigmoid colon cancer. HIPEC with oxaliplatin, 5-fluorouracil and mitomycin C were given as adjuvant therapy. Subsequently, intestinal obstruction developed at 15 months postoperatively, and the patient was hospitalized. Abdominal computed tomography showed a dilated small intestine enveloped by a thickened membrane. We found no evidence of peritoneal recurrence, but exploratory surgery revealed EPS, probably caused by HIPEC. We peeled the capsule off of the intestine. The patient’s postoperative course was uneventful, and sufficient nutritional intake after surgery was noted. Seven months after surgery, he is well with no recurrence.

**Conclusion:**

The surgical treatment via peritonectomy and enterolysis for postoperative EPS appears safe and effective. A diagnosis of EPS should be considered when intestinal obstruction does not show improvement with conservative treatment in patients who have undergone HIPEC, provided the possibility of peritoneal cancer recurrence is excluded.

## Background

The use of cytoreductive surgery combined with hyperthermic intraperitoneal chemotherapy (HIPEC) is a novel approach that offers improved survival after peritoneal surface malignancy, including colorectal peritoneal metastases (PM) [1-3]. HIPEC is also an attractive option for the treatment of patients at high risk of developing PM after surgical management of advanced colorectal cancer [4,5]. We previously conducted a phase I clinical trial of the safety and efficacy of HIPEC for colorectal cancer in patients who were at high risk of developing PM after surgery [4]. However, HIPEC is associated with an increased risk of surgical complications and toxicity. Canda et al. described the morbidity and mortality rates after HIPEC [6]. Complications of such therapy may include nephrotoxicity, hemotoxicity, postoperative hemorrhage, anastomotic leakage, intestinal perforation, and wound complications. Awareness and early recognition of the surgical complications and toxicity of this approach may help reduce mortality rates.

Encapsulating peritoneal sclerosis (EPS) is a rare surgical complication that can occur after intraperitoneal treatment. It is also a serious and potentially fatal complication of continuous ambulatory peritoneal dialysis (CAPD) [7]. Some reports have described EPS occurring after intraperitoneal chemotherapy (IPC), and EPS is considered as a delayed complication of IPC or HIPEC [7,8]. The present report describes a case of surgically treated EPS that probably occurred as a complication of HIPEC.

## Case presentation

**Figure 1:**
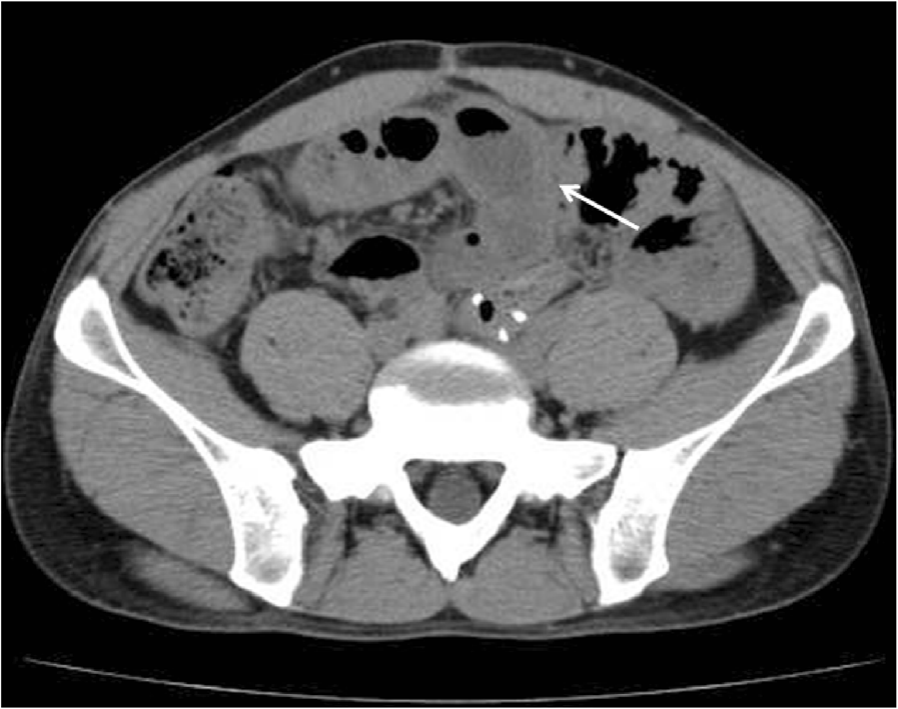
**Abdominal computed tomography shows a dilated and filled small intestine enveloped by a thickened membrane (*arrow*).**

**Figure 2:**
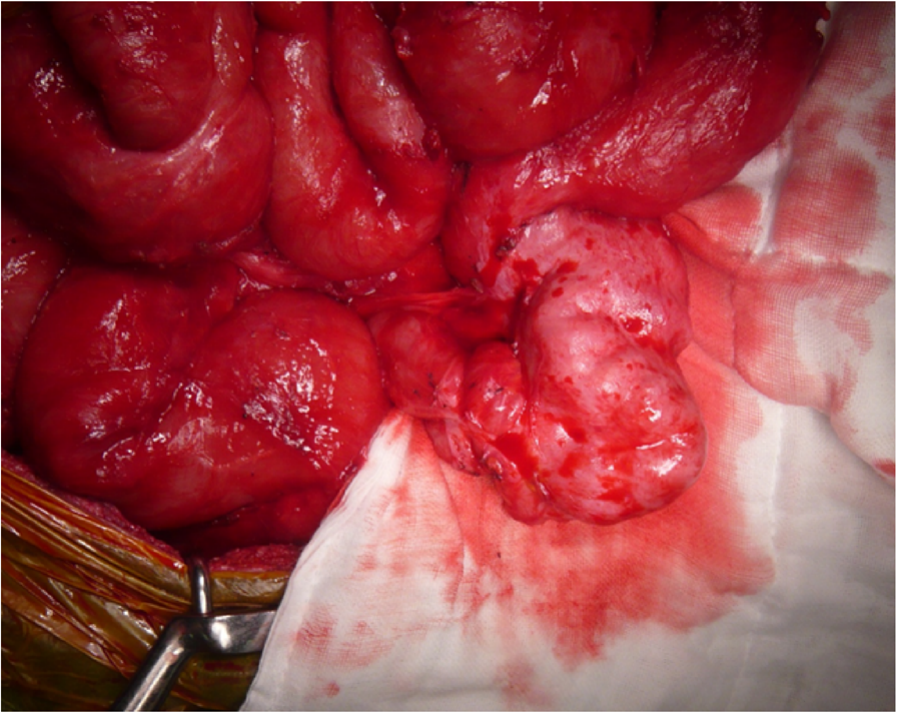
**Operative findings showed small intestine covered by white and fibrous membrane.** The small intestine is partially covered by a white membrane and looks like a cocoon. The fibrous membrane is peeled off to release the small intestine.

**Figure 3:**
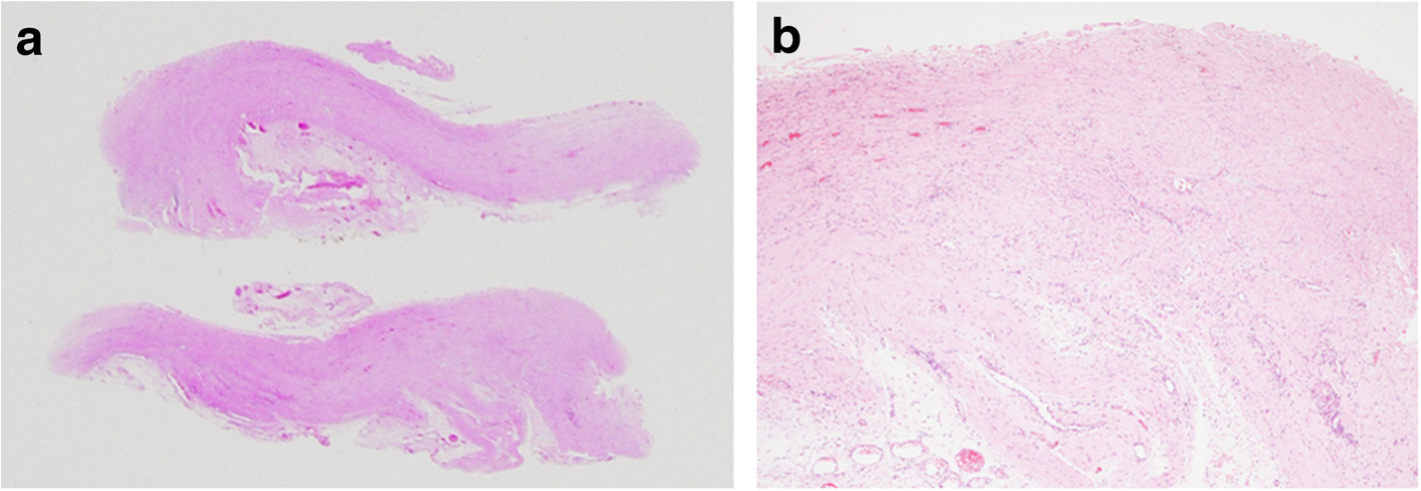
**Histological features of the case. (a)**, **(b)** Histological findings of the thickened membrane show proliferation of fibroconnective tissue and inflammatory infiltrates.

A 39-year-old man with serosal invasive advanced sigmoid colon cancer underwent surgery at Shiga University of Medical Science Hospital. Sigmoidectomy with regional lymph node dissection followed by reconstruction with stapled functional end-to-end anastomosis was performed. HIPEC was subsequently delivered as described previously [4]. Briefly, the laparotomy incision was closed until an 18-cm opening. An Alexis Wound Retractor (Applied Medical Resource, CA, USA) and plastic cylinder were used to make a waterbath space for HIPEC. A perfusate of 5 L saline solution (Otsuka Pharmaceutical Factory, Tokushima, Japan) was circulated at flow rate of 500–750 mL/min by using the CP-3000 system (Tonokura, Tokyo, Japan). After the temperature of the perfusate reached 42-43°C, 10 mg of mitomycin-C, 1000 mg of 5-fluorouracil, and 200 mg of oxaliplatin was added. HIPEC was performed for 30 minutes after the addition of the anti-tumor drugs. We stirred the perfusate manually and extensively to strictly maintain the temperature of the abdominal perfusate between 42 and 43°C. Histological examination revealed moderately differentiated adenocarcinoma without lymph node metastasis. The disease was classified as stage II, T4N0M0 according to the TNM classification. The number of dissected lymph nodes was 10 at this operation. There were no postoperative complications, and 4 days after the operation, he started oral intake. He was discharged from hospital on the 8th postoperative day. He received adjuvant chemotherapy with oral capecitabine administration for 3 months after surgery.Approximately 15 months after surgery, the patient presented to our hospital complaining of nausea, abdominal distention, and abdominal pain. His body weight had decreased from 60 kg at the time of initial surgery to 50 kg. Levels of the tumor markers, carcinoembryonic antigen and carbohydrate antigen 19–9, were within normal range. Abdominal x-ray showed increased intestinal gas and gas-fluid levels. He was admitted to the hospital with a diagnosis of adhesive intestinal obstruction. Abdominal computed tomography (CT) scan showed a dilated small intestine enveloped by a thickened membrane (Figure 1). After admission, we diagnosed as EPS based on these data. He was treated with decompression via long intestinal tube and with intravenous betamethasone administration. After 3 days, intestinal obstruction did not improve with such conservative treatments. Laparotomy was performed, showing most of the small intestine folded and extensively enveloped by a thickened membrane, with the appearance of a cocoon (Figure 2). No peritoneal nodule was found, and cytological examination of the peritoneal washing did not show any cancer cells. No signs of peritoneal recurrence were found. Incision of the thickened membrane surrounding the small intestine confirmed that the serosa of the small intestine was normal without any cancer invasion. The procedure was performed easily, except in the terminal ileum where there was tight adhesion and round fibrous stenosis. The round fibrous thickening of the small intestine was alleviated by incising the surrounding thickened membrane. There were no postoperative complications, including recurrence of intestinal obstruction. Seven days after the operation, he started oral intake. He was discharged from hospital on the 18th postoperative day. Histological findings of the thickened membrane showed proliferation of fibroconnective tissue and inflammatory infiltrates (Figure 3a, b). No histological signs of peritoneal recurrence were found. Seven months after surgery, he is well, with no radiological signs or laboratory evidence of recurrence, and his body weight has increased back to that observed in the preoperative state.

This report described a rare case of EPS after HIPEC that was successfully treated via the surgical approach. EPS causes intestinal obstruction, because the small intestine is enveloped by a secondary thick fibrosis. Several report showed the morbidity rates after HIPEC were 20.8-53.3% [6]. Major complications may include nephrotoxicity, hemotoxicity, postoperative hemorrhage, anastomotic leakage, intestinal perforation, and wound complications [6]. EPS is a rare surgical complication that can occur after HIPEC. The cause is still unclear, although several factors have been implicated, including CAPD treatment for chronic renal failure and a past history of peritonitis or cirrhotic ascites [9]. Only one report describes the occurrence of EPS as a delayed complication of HIPEC [7].

Previously, 9 cases of EPS after IPC and 1 case of EPS after HIPEC were reported (Table 1) [7,8,10,11]. Most of the cases developing EPS underwent intraperitoneal chemotherapy including platinums. One of the possible causes of EPS in patients undergoing CAPD is related to the low pH of the dialysate [7]. The incidence of EPS decreases in response to agents that neutralize the pH of the dialysate [7]. In our method, the optimal pH for Oxaliplatin ranges from approximately 4.0 to 7.0 [4], and this acidity might contribute to the development of EPS.

**Table 1:** Reported cases of encapsulating peritoneal sclerosis after intraperitoneal chemotherapy treated by the surgical approach

**No**	**Authors**	**Year**	**Age (years)**	**Gender**	**Disease**	**Agent**	**Hyperthermia**	**EPS after initial surgery**	**Treatment for EPS**	**Peritoneal recurrence**	**Recurrence of EPS**	**Survival after initial surgery**
1	Braly [11]	1986	Unknown	Female	Ovarian cancer	Cisplatin, 5-fluorouracil	No	Unknown	Laparotomy	No	Unknown	Unknown
2	Braly [11]	1986	Unknown	Female	Ovarian cancer	Cisplatin, 5-fluorouracil	No	Unknown	Laparotomy	No	Unknown	Unknown
3	Braly [11]	1986	Unknown	Female	Ovarian cancer	Cisplatin, 5-fluorouracil	No	Unknown	Laparotomy	No	Unknown	Unknown
4	Vlasveld [10]	1992	44	Male	Mesothelioma	Metoxantrone	No	24 months	Surgery	No	No	Alive, 48 months
5	Atiq [8]	1993	Unknown	Unknown	Gastric cancer	Cisplatin, 5-fluorouracil	No	4 to 17.5 months	Surgery	No	No	Unknown
6	Atiq [8]	1993	Unknown	Unknown	Gastric cancer	Cisplatin, 5-fluorouracil	No	4 to 17.5 months	Surgery	No	No	Unknown
7	Atiq [8]	1993	Unknown	Unknown	Gastric cancer	Cisplatin, 5-fluorouracil	No	4 to 17.5 months	Surgery	No	No	Unknown
8	Atiq [8]	1993	Unknown	Unknown	Gastric cancer	Cisplatin, 5-fluorouracil	No	4 to 17.5 months	Surgery	No	Yes	Unknown
9	Atiq [8]	1993	Unknown	Unknown	Gastric cancer	Cisplatin, 5-fluorouracil	No	4 to 17.5 months	Laparotomy	Yes	Unknown	Unknown
10	Aihara [7]	2003	47	Female	Gastric cancer	Cisplatin, mitomycin-C	Yes	6 months	Surgery	No	No	Alive, 21 months
11	Our case	2014	39	Male	Colon cancer	Oxaliplatin, 5-fluorouracil, mitomycin-C	Yes	16 months	Surgery, oral steroid therapy	No	No	Alive, 24 months

*Abbreviation:EPS* encapsulating peritoneal sclerosis.

An intraperitoneal temperature of 42-43°C is necessary to obtain the maximal therapeutic effects of HIPEC [7]. Previous reports have described EPS occurred even among patients treated by normothermia [8,10]. Shindo et al. reported that peritoneal damage was not dissimilar between patients given HIPEC and those given continuous normothermic peritoneal perfusion using 37.0°C perfusate [12]. However, hyperthermia induced intracellular acidosis [13] and may accelerate instability in intracellular pH. Thus, we consider hyperthermia may be a cause of EPS.

Steroid therapy is considered to be effective for preventing EPS, because the “second hit” in development of EPS may be related to various cytokines. The second hit theory whereby dialysis fluids contribute to peritoneum damage. Cases of peritoneal dialysis-related EPS have responded favorably to corticosteroid therapy alone [9]. In our case, however, steroid therapy was not effective.

In our case, incision and resection of the sclerosing peritoneum resulted in improvement of stenosis without postoperative complications. It is important that the enterolysis is performed without damaging the encapsulated intestine. The overall morbidity rate of surgery for EPS is reported as approximately 60% [14]. However, Kawanishi et al. reported successful treatment of 18 patients with CAPD-related EPS and suggested that peritonectomy and enterolysis for EPS was a useful therapeutic method [15]. They emphasized the critical role of early diagnosis of EPS as well as determination of the therapeutic strategy according to the disease stage. In three patients, EPS was relieved by steroid therapy. In 15 patients, EPS was relieved by total intestinal enterolysis. These patients had satisfactory operative outcomes and returned to their previous activity. Ulmer et al. reviewed 26 patients with EPS who underwent peritonectomy and enterolysis [16]. They suggested that this treatment option could be performed with low mortality and acceptable morbidity. Thus, peritonectomy and enterolysis are considered to be appropriate methods for EPS induced by HIPEC in a manner similar to that for EPS induced by CAPD.

## Conclusion

Preoperative distinction among adhesion, EPS, or PM can be difficult when small bowel obstruction occurs in patients who have received HIPEC. A diagnosis of EPS should be considered if intestinal obstruction does not show improvement with conservative treatment, provided that the possibility of PM is excluded. Early laparotomy and resection of thickening fibrosis are important to improve the patient’s quality of life. In addition, ongoing efforts to increase the feasibility and safety of intraperitoneal chemotherapy are warranted.

## Consent

Written informed consent was obtained from the patient for publication of this Case report and any accompanying images. A copy of the written consent is available for review by the Editor of this journal.

## Abbreviations

EPS: Encapsulating peritoneal sclerosis; HIPEC: Hyperthermic intraperitoneal chemotherapy; PM: Peritoneal metastasis; CAPD: Continuous ambulatory peritoneal dialysis; IPC: Intraperitoneal chemotherapy; CT: Computed tomography.

## Competing interests

None of the authors have a conflict of interest or have received financial support.

## Authors’ contributions

KT drafted the manuscript, and conducted a literature search. HS, TS and HO conducted a literature search and contributed to drafting the manuscript. MI evaluated histopathological examination. HS, TS, HO, EM, YE and TT performed the operation and reviewed the manuscript and gave final approval for publication. All authors read and approved the final manuscript.

## Pre-publication history

The pre-publication history for this paper can be accessed here:

http://www.biomedcentral.com/1471-2482/14/57/prepub
